# Supplementary material for: Polyketide synthase gene clusters in the ericoid mycorrhizal fungus Oidiodendron maius: genome-wide prediction, expression profiling, and functional characterization of knockout mutants
Source: Front Plant Sci. 2026 Sep 16;17:1924430. doi: 10.3389/fpls.2026.1924430 (PMC13623903; doi:10.3389/fpls.2026.1924430)
Supplement: Supplementary file 1 [file SupplementaryFile1.zip › Supplementary Materials/Supplementary Folder 1.zip!/Oidma1_AssemblyScaffolds_Repeatmasked/knownclusterblast/cluster2/jgi.p_Oidma1_46805_mibig_hits.html]

| MIBiG Protein | Description | MIBiG Cluster | MiBiG Product | % ID | % Coverage | BLAST Score | E-value |
| --- | --- | --- | --- | --- | --- | --- | --- |
| CCH26291.1 | putative\_O-methyltransferase | BGC0001158\_c2 | Bikaverin\_biosynthetic\_gene\_cluster | 41 | 101.805869074 | 336 | 3.8e-92 |
| CCT67993.1 | bikaverin\_cluster-O-methyltransferase | BGC0000030 | Bikaverin\_biosynthetic\_gene\_cluster | 41 | 96.1625282167 | 333 | 4.2e-91 |
| ABC79591.2 | cercosporin\_toxin\_biosynthesis\_protein | BGC0001247\_c2 | Cercosporin\_biosynthetic\_gene\_cluster | 41 | 97.2911963883 | 307 | 2.5e-83 |
| CCE67071.1 | methyltransferase | BGC0001242 | Fusarubin\_biosynthetic\_gene\_cluster | 40 | 84.8758465011 | 267 | 2.2e-71 |
| AAS90018.1 | OmtA | BGC0000007 | Aflatoxin\_biosynthetic\_gene\_cluster | 27 | 93.6794582393 | 129 | 1.2e-29 |
| BAE71329.1 | o-methyltransferase\_A | BGC0000004 | Aflatoxin\_biosynthetic\_gene\_cluster | 28 | 78.7810383747 | 128 | 2.1e-29 |
| AAS90087.1 | OmtA | BGC0000010 | Aflatoxin\_biosynthetic\_gene\_cluster | 26 | 93.6794582393 | 126 | 6e-29 |
| AAS90065.1 | OmtA | BGC0000009 | Aflatoxin\_biosynthetic\_gene\_cluster | 28 | 77.4266365688 | 126 | 1e-28 |
| AAS90104.1 | OmtA | BGC0000006 | Aflatoxin\_biosynthetic\_gene\_cluster | 26 | 93.6794582393 | 125 | 1.7e-28 |
| AAS90041.1 | OmtA | BGC0000008 | Aflatoxin\_biosynthetic\_gene\_cluster | 27 | 93.6794582393 | 125 | 1.7e-28 |
| ACN64847.1 | PokMT3 | BGC0001061 | Polyketomycin\_biosynthetic\_gene\_cluster | 35 | 34.085778781 | 84 | 4.5e-16 |
| AKD43502.1 | O-Methyltransferase | BGC0001409 | Dutomycin\_biosynthetic\_gene\_cluster | 33 | 34.085778781 | 77 | 3.2e-14 |
| CAF31570.1 | putative\_O-methyltransferase | BGC0000705 | Kanamycin\_biosynthetic\_gene\_cluster | 31 | 48.0812641084 | 76 | 7.1e-14 |
| CAF60515.1 | putative\_O-methyltransferase | BGC0000704 | Kanamycin\_biosynthetic\_gene\_cluster | 31 | 48.0812641084 | 76 | 7.1e-14 |
| BAE95598.1 | putative\_O-methyltransferase | BGC0000703 | Kanamycin\_biosynthetic\_gene\_cluster | 31 | 48.0812641084 | 76 | 7.1e-14 |
| AAA67518.1 |  | BGC0000275 | Tetracenomycin\_biosynthetic\_gene\_cluster | 27 | 48.3069977427 | 76 | 1.2e-13 |
| AHE14647.1 | O-methyltransferase | BGC0001336 | Erdasporine\_biosynthetic\_gene\_cluster | 32 | 43.1151241535 | 72 | 1e-12 |
| AHZ61883.1 | Lom49 | BGC0000240 | Lomaiviticin\_biosynthetic\_gene\_cluster | 28 | 63.6568848758 | 70 | 6.7e-12 |
| CCC55909.1 | O-methyltransferase | BGC0000973 | Collismycin\_A\_biosynthetic\_gene\_cluster | 27 | 74.0406320542 | 70 | 6.7e-12 |
| AAQ08925.1 | putative\_o-methyltransferase | BGC0000224 | Fredericamycin\_biosynthetic\_gene\_cluster | 26 | 75.39503386 | 69 | 1.5e-11 |
| ABP54665.1 | O-methyltransferase,\_family\_2 | BGC0000241 | Lomaiviticin\_biosynthetic\_gene\_cluster | 27 | 66.3656884876 | 68 | 2.5e-11 |
| ACB47081.1 | DynO6 | BGC0001060 | Dynemicin\_biosynthetic\_gene\_cluster | 27 | 43.7923250564 | 67 | 4.3e-11 |
| AAA83421.1 | RdmB | BGC0000265 | Rhodomycin\_biosynthetic\_gene\_cluster | 31 | 34.762979684 | 67 | 4.3e-11 |
| AEE65480.1 | putative\_O-methyltransferase | BGC0000223 | Fluostatin\_biosynthetic\_gene\_cluster | 32 | 33.4085778781 | 64 | 4.8e-10 |
| KDN80042.1 | methyltransferase | BGC0001074 | Cosmomycin\_D\_biosynthetic\_gene\_cluster | 26 | 58.9164785553 | 63 | 6.2e-10 |
| ADI24956.1 | GsfD | BGC0000070 | Griseofulvin\_biosynthetic\_gene\_cluster | 28 | 41.9864559819 | 60 | 5.3e-09 |
| AAM70343.1 | CalO1 | BGC0000033 | Calicheamicin\_biosynthetic\_gene\_cluster | 27 | 74.0406320542 | 60 | 5.3e-09 |
